# A new application of *Trichoderma asperellum* as an anopheline larvicide for eco friendly management in medical science

**DOI:** 10.1038/s41598-018-37108-2

**Published:** 2019-02-01

**Authors:** Dipanjan Podder, Swapan Kr. Ghosh

**Affiliations:** Molecular Mycopathology Lab., Biological Control and Cancer Research Unit, PG Department of Botany, Ramakrishna Mission Vivekananda Centenary College (Autonomous), Rahara, Kolkata, 700118 W.B. India

## Abstract

Microfungal applications are increasing daily in the medical science. Several species of *Trichoderma* are widely used in agricultural fields as biological control and plant growth promoting agents. The application of *Trichoderma asperellum* as an entomopathogenic fungus against the *Anopheles* mosquito, a vector of malaria, is a novel control approach. Controlling malaria with eco-friendly management practices is an urgent need. We isolated three *T. asperellum* from different natural sources using serial dilution and mosquito baiting techniques. The fungi were identified on the basis of phenotypical and molecular characteristics. The fungi were grown in different natural media to examine spore production ability and the fungal spore suspensions were applied to the anopheline larvae to determine their larvicidal activity *in vitro*. We investigated the efficacy of crude ME (methanolic extract) and different methanolic fractions (MFs) of the fungal extracts against anopheline larvae. Methanolic Fraction 8 (MF8) exhibited the strongest larvicidal activity. A GC-MS analysis of MF8 and a Chemolibrary search were performed to identify the active agents in the fungal extracts. Among the three isolates of *T. asperellum*, the TaspSKGN2 isolate showed the lowest LD_50_ (2.68 × 10^7^ conidia/mL) and LT_50_ values (12.33 h). The crude ME exhibited LD_50_ values of 0.073 mg/mL and LT_50_ values of 11.33 h. MF8 showed LD_50_ values of 0.059 mg/mL and LT_50_ values of 8.57 h. In GC-MS study of MF8, 49 compounds were found. Among these, seven compounds (2,3-di hydro thiopene, p-cymene, alpha-pinene, hexadecanoic acid, 8-methyl quinoline, (Z,Z)-9,12-octa decadienoic acid, methyl ester, 2,3-dihydro-3,5-dihydroxy-6-methyl-4H-Pyran-4-one-) with high abundance were found to have insecticidal efficacy by a literature survey. We detected a reduction in the phenoloxidase content inside the cuticle and hemolymph of the anopheline larvae after a few hours of interaction with ME (0.073 mg/mL). Thus *Trichoderma asperellum* has new applications for the control of *Anopheles* spp. malaria vectors.

## Introduction

Mycological research is producing a new wave of applications for microfungi in different areas of environmental and health management that will likely boost the field of mycology. *Trichoderma* spp. belong to the phylum Ascomycota, sub phylum Pezizomycotina, class Sordariomycetes, order Hypocreales, and family Hypocreaceae and are well known for their ability to attack other microbes by producing antibiotics and other extracellular enzymes; consequently *Trichoderma* spp. have been utilized as biocontrol agents against plant pathogens for approximately 70 years^1^. *Trichoderma* spp. are widely used in agriculture, both for disease control and to increase plant yields^2^. Compost made with agricultural waste and the *Trichoderma asperellum* strain suppresses *Rhizoctonia solani* in cucumber seedlings^3^. *Trichoderma asperellum* was reported as a potent bio control agent against seed-borne rice diseases in a previous study^4^. In our laboratory *Trichoderma longibrachiatum* was reported to be entomopathogenic against *Leucinodes orbonalis*, a major pest of eggplant^5^. As per this report, the effectiveness of the entomopathogen was comparable to the chemical insecticide malathion. In this present study, the anopheline larvicidal efficacy of *T. asperellum* is established. To the best of our knowledge, this is the first report of the anopheline larvicidal potential of *T. asperellum*.

Malaria, a mosquito borne infectious disease in humans and other animals, is caused by parasitic Apicomplexan protozoans belonging to the genus *Plasmodium*. Previous research has established that most human deaths are caused by *P. falciparum* but *P. vivax*, *P. ovale*, and *P. malariae* generally cause a milder form of malaria^6^. The mosquito genus, *Anopheles* belongs to the order *Diptera*, family *Culicidae*, subfamily *Anophelinae*. Of the 56 ubiquitous species in India, thirteen are malarial vectors. According to the WHO (World Health Organization) malaria is arguably the most serious mosquito vector borne disease globally. Data presented in the World Malaria Report^7^ showed that over 70% of India’s population is at risk of malaria infection and approximately 31 × 10^7^ people are at the “highest risk” of becoming infected. India has over 10 × 10^7^ suspected malaria cases but only 15.9 × 10^5^ could be confirmed in 2010. On average, 40.297 × 10^3^ Indians die of the mosquito-borne disease every year. In 2013, 0.88 million cases of malaria were recorded. According to the World Malaria Report 2014, 22% (275.5 m) of India’s population lives in high transmission (>1 case per 1000 individuals) areas, 67% (838.9 m) live in low transmission (0–1 cases per 1000 individuals) areas and 11% (137.7 m) live in malaria-free (0 cases) areas. In 2015, there was an estimated 4.38 × 10^5^ malaria deaths worldwide^7^.

In general four pesticides: Scourge, anvil, permethrin and malathion are applied against mosquito. Scourge (pipernyl butoxide) and permithrin (pyrethroid) have been identified by the US EPA (Environment Protection Agency) as possible human carcinogen^8^. Anvil or Sumithrin (pipernyl butoxide) promotes cancer in reproductive organ (breast and prostate)^9^. Malathion (diethyl 2-dimethoxyphosphinothioylsulfanylbutanedioate) has also been denoted as low level carcinogen by the EPA^8^ and their bioaccumulation in the environment is hazardous. The already alarming number of deaths caused by malaria is increasing, which is caused partly by the increase in mosquito resistance to chemical insecticides. Chemical control, therefore, cannot be a solution. Instead the biological control of mosquito vectors can be an alternative, eco-friendly, green approach. Although many biological control agents such as *Bacillus thuringensis* and *Bacillus sphaericus* have been found to be effective against mosquitoes, resistance has already started to grow in mosquito against these larvicides^10^. The eradication of malarial vectors through new biological control agents with better efficacy, therefore, should be a priority for modern day researchers.

Spores and metabolites of entomopathogenic fungi have been reported as larvicides/biocontrol agents against mosquitoes by previous workers^11–14^. The search of entomopathogens with good efficacy, viability, ease of grow and cost effectiveness is still ongoing active areas of research, *Beauveria bassiana* has been reported to be an effective bio control agent against mosquito larvae^15^. Earlier studies have indicated the entomopathogenic potential of ascomycetous fungi (*Metarhizium anisopliae* and *B. bassiana*) as insecticides^16^. Furthermore, the entomopathogenic effects of *Trichophyton ajelloi* against *Anopheles stephensi* have been observed^17^. Extracellular metabolites of *Trichophyton ajelloi*, *Chrysosporium tropicum* and *Lagenidium giganteum* have been found to have larvicidal activity when leveraged against *Anopheles stephensi*^18–21^. In this experiment indigenous strains of *Trichoderma asperellum* were isolated from soils of several districts (Malda, Nadia, Hooghly, Birbhum, North & South 24 Parganas) of West Bengal and their spores, crude ME and ME fractions applied against the larvae of *Anopheles* spp. Here, we continue the search for potent fungal strains with better efficacies than those reported in earlier studies and aim to find effective indigenous fungi that can be used in their endemic regions to control anopheline larvae (despite the utilization of a fungal strain from outside of West Bengal). To the best of our knowledge, this is the first report to screen *Trichoderma asperellum* as a bioinsecticide against mosquito larvae.

## Results

### Isolation and Phenotypic identification of the isolated fungus from different zones of West Bengal

**Figure 1:**
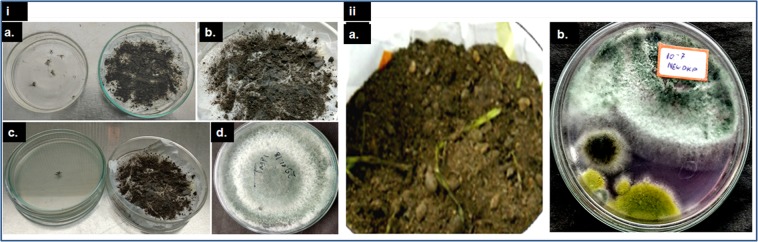
Isolation of *T. asperellum* from soil. (**i**) Mosquito baiting technique a. Inoculation of dead adult mosquito b. Growth of fungus upon mosquitoes c. Transfer of fungi infected mosquitoes to a petridish containing PDA d. Growth of fungus in PDA medium. (**ii**) Soil dilution technique a. Soil sample b. Growth of several fungal colonies in the soil dilution plate.

**Figure 2:**
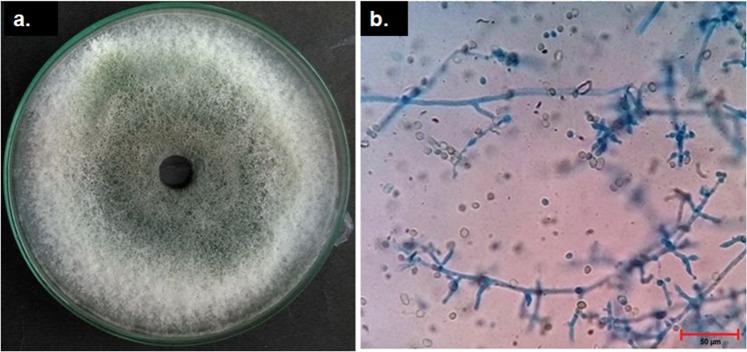
Phenotypical characteristics of *T. asperellum*. (**a**) Culture plate. (**b**) Microscopic view of the lactophenol cotton blue mount of *T. asperellum* (TaspSKGN2) (10x).

The fungus was isolated from soils of different zones of West Bengal (Table 1) by mosquito baiting and soil dilution technique (Fig. 1). The fungus was identified as *Trichoderma asperellum* (TaspSKGN2) on the basis of cultural and microscopic characteristics (Fig. 2) reported in the literature^22–26^. In terms of the geographical distribution we noted that isolates of *Trichoderma asperellum* were found mainly in the soil samples from Hooghly, Malda and South 24 Parganas in the state of West Bengal.

**Table 1:** Isolates of *T. asperellum* from natural sources from different districts of West Bengal with the collection date, time and temperature.

Sr No.	Isolate	Sample	Districts	Date	Time	Temperature
1	*Trichoderma asperellum* (TaspSKGKN1)	Cultivated soil	Hooghly	07/07/2017	4:30 pm	38 °C
2	*Trichoderma Asperellum* (TaspSKGKN3)	Field soil	Maldah	28/07/2017	1:30 pm	32 °C
3	*Trichoderma Asperellum* (TaspSKGN2)	Vermicompost/Soil	South 24 Parganas	18/09/2017	11:30 am	29 °C

### Preliminary screening of the larvicidal efficacy and the selection of most potent isolate

Three fungal isolates were preliminarily screened for activity against anopheline larvae (data not presented) and TaspSKGN2 performed the best larvicidal efficacy. So, TaspSKGN2 was selected for further experiments.

### Molecular identification of the fungus

**Figure 3:**
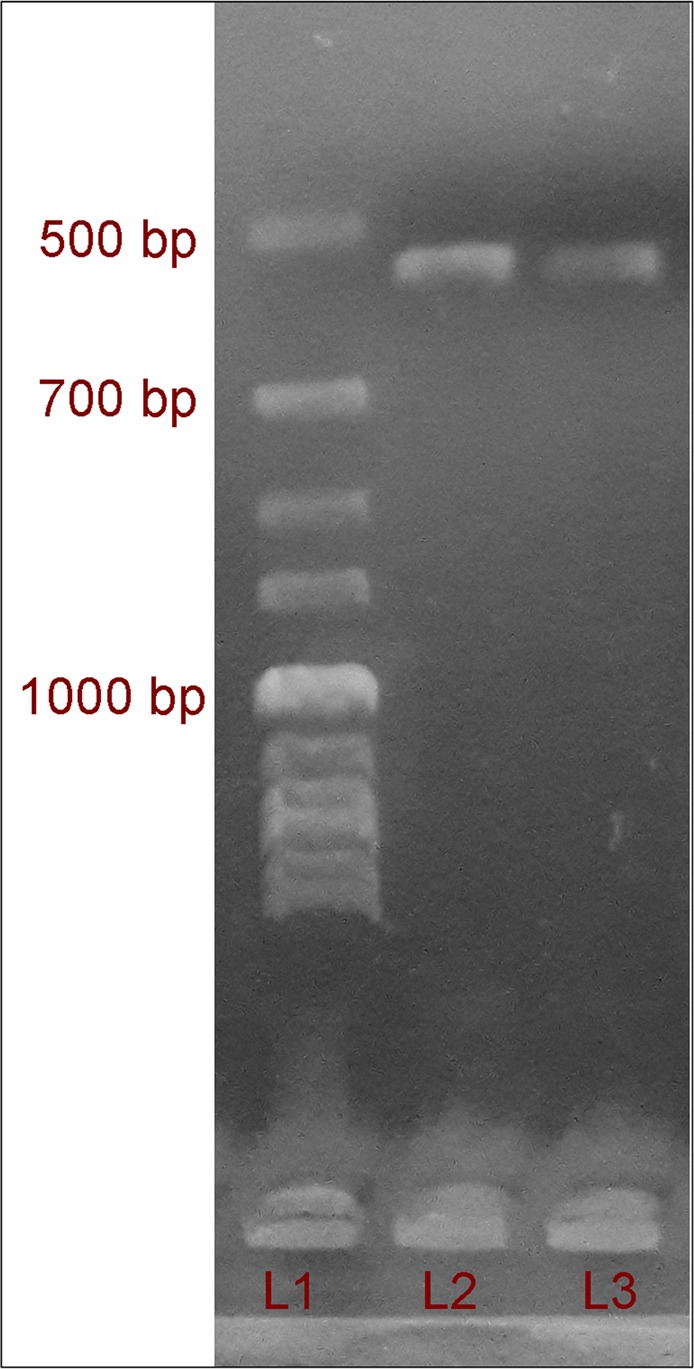
Agarose gel electrophoresis: band pattern of PCR amplified genomic DNA under UV transilluminator. L1. DNA Ladder, L2 & L3. Sample (amplicon); Bands of DNA amplicon near 500 bp.

After extraction of genomic DNA from *Trichoderma* sp. by using a modified CTAB method^27^, the ITS region (Internal transcribed Spacer) including 5.8S of the genomic DNA was amplified by Thermal cycler using PCR (Fig. 3). The amplified ITS1-5.8S-ITS2 region of the genomic DNA was sequenced at Sci-Genome Laboratory, Kerala, India. The nucleotide sequences obtained were subjected to BLAST in the NCBI database^28^; based on query coverage (97%) and the identity score (99%) of sequence homology search, fungal strain/isolate was identified as *Trichoderma asperellum*. First, we identified our fungal strain as *Trichoderma asperellum* phenotypically. Molecular identification also validated our fungal isolate as *Trichoderma asperellum*. The nucleotide sequence of the isolate (TaspSKGN2) was submitted to GenBank (NCBI) under the accession number MG719999.1.

### Phylogenetic tree analysis

**Figure 4:**
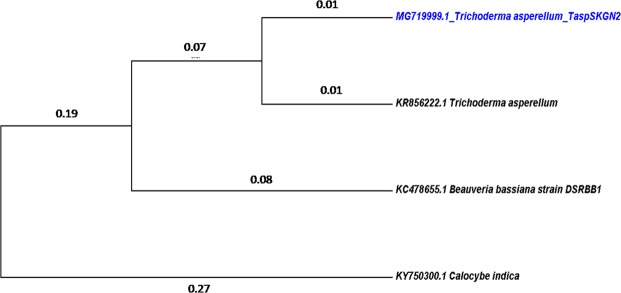
Evolutionary relationship of taxa.

A phylogenetic tree was constructed using the UPGMA method (Unweighted Pair Group Method with Arithmatic Mean)^29^ by MEGA 7 software to determine the particular evolutionary position of our isolated fungus (Fig. 4). From our constructed phylogenetic tree it can be inferred that our isolate is closely related to another *T. asperellum* (Accession no. KR856222.1) and it exhibits distant relationship with *B. bassiana* (Accession no. KC478655.1) and more distant relationship with *C. indica* (Accession no. KY750300.1) based on evolutionary time scale.

### Effects of different natural media on the mass production of spore of *T. asperellum* (TaspSKGN2)

**Figure 5:**
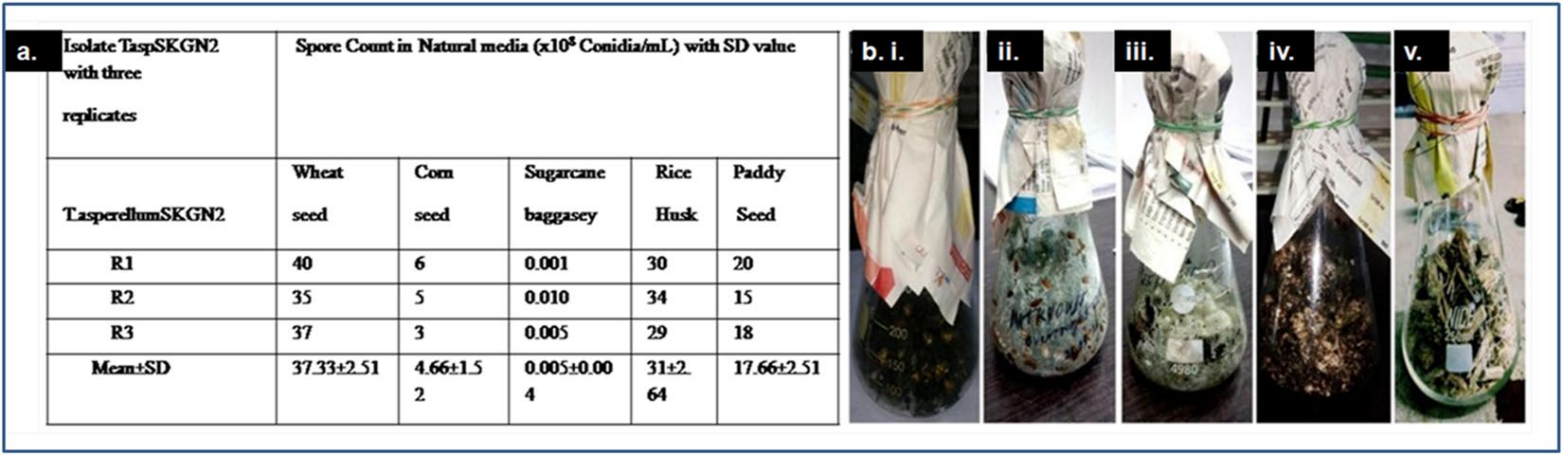
Mass spore production of *T. asperellum* (TaspSKGN2). (**a**) Spore production (mean ± SD) by *Trichoderma asperellum* (TaspSKGN2) in different natural media from three replicates (tabular form). (**b**) Photographs of mass spore production in different natural media (after 15 d of inoculation): i. Corn medium, ii. Paddy seed medium, iii. Rice husk medium, iv. Wheat medium, v. Sugarcane bagasse medium.

Five types of natural media i.e. wheat grain medium, corn grain medium, sugarcane bagasse medium, rice husk medium and paddy seed medium were used for mass spore production (Fig. 5). The amount of spores that *T. asperellum* produced in these natural media was calculated after fifteen days of fungal inoculation. A comparative study of the effects of these media on spore production is presented in Fig. 5. Among these five natural media, wheat grain medium supported maximum spore production (37.33 × 10^8^ conidia/mL) of *T. asperellum* (TaspSKGN2) followed by rice husk medium (31 × 10^8^ conidia/mL), paddy seed medium (17.66 × 10^8^ conidia/mL), corn grain medium (4.66 × 10^8^ conidia/mL) and Sugarcane bagasse medium (0.005 × 10^8^ conidia/mL) after 15 d of fungal inoculation.

### Anopheline larvicidal activity of the spore suspensions of *T. asperellum* (TaspSKGN2)

Different spore concentrations (10^1^–10^8^ conidia/mL) of the *T. asperellum* (TaspSKGN2) isolate were applied to the anopheline larvae. At a 10^1^ conidia/mL spore concentration of TaspSKGN2, no larval death was found within 24 h. Larval mortality (%) was observed at 48 h (8.33 ± 2.88) and gradually increased with time i.e., at 72 h, 15% larval mortality was noted for that concentration. When the 10^2^ conidia/mL spore concentration was applied, larval mortality was observed within 24 h i.e., 3.33 ± 2.88 and the mortality gradually increased with time following the same trend i.e., at 48 h (11.66 ± 7.63) and 72 h (15.00 ± 5.00), the death percentages increased. At a 10^3^ conidia/mL spore concentration, the larval mortalities (%) were 10.00 ± 5.00, 25.00 ± 5.00 and 28.33 ± 7.63 after 24, 48 and 72 h of observation respectively. At a 10^4^ conidia/mL spore concentration, the death percentages of larvae were 20.00 ± 5.00, 26.66 ± 5.77 and 35.00 ± 5.00 at 24, 48 and 72 h of observation respectively. At a 10^5^ conidia/mL concentrations after 24, 48 and 72 h, the larval mortalities (%) were found to be 28.33 ± 7.63, 41.66 ± 7.63 and 48.33 ± 10.40 respectively. At a 10^6^ conidia/mL spore concentration, more than 50% larval mortality was found after 48 h of treatment i.e., 55 ± 5.00 and more than 60% mortality was found after 72 h of treatment i.e. 63.33 ± 7.63. More than 50% larval mortality was found within 24 h for the 10^7^ conidia/mL treatment i.e. 53.33 ± 5.77, and the larval mortalities were 65.00 ± 5.00 and 85.00 ± 5.00 at 48 and 72 h of treatment, respectively. In the case of the 10^8^ conidia/mL spore concentration, 80% of larval deaths were noted within 24 h and 96.66 ± 5.77 and 100% mortality were observed at 48 and 72 h, respectively. It was noted that with each spore concentration, mortality increased with increasing time and mortality also increased with increasing spore concentrations. LD_50_ value of TaspSKGN2 was calculated using regression analysis with MS EXCEL. The isolate exhibited an LD_50_ value of 2.68 × 10^7^ conidia/mL (Table 2).

**Table 2:** Calculation of the LD_50_ value of the TaspSKGN2 spore suspensions with MS EXCEL.

Isolate	Regression equation	R^2^ value	LD_50_ value (conidia/mL)
TaspSKGN2	Y = 6.7687 × 10^−7^X + 31.84	0.624	**2.68 × 10** ^**7**^

The LD_50_ dose (2.68 × 10^7^ conidia/ml**)** of TaspSKGN2 was applied to anopheline larvae to determine the lethal time. Every set was run in triplicates. More than 60% of the larval deaths were noted after 24 h of treatment i.e., 66.66 ± 2.88. Mortality increased with increasing time. More than 70% of mortality was observed at 48 h i.e., 76.66 ± 5.77 and approximately 90% of larval mortality was observed after 72 h of treatment i.e. 88.33 ± 2.88. The LT_50_ value of TaspSKGN2 was calculated using regression analysis with MS EXCEL. The isolate exhibited an LT_50_ value of 12.33 h (Table 3).

**Table 3:** Calculation of the LT_50_ value of TaspSKGN2 using regression analysis with MS EXCEL.

Isolate	Regression equation	R^2^ value	LT_50_ value (h)
TaspSKGN2	Y = 0.45X + 55.55	0.998	**12.33**

**Figure 6:**
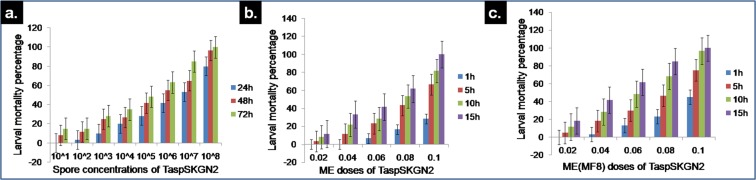
Graphical representation of the mortality percentages of anopheline larvae exposed to spore, ME and MF8 doses of TaspSKGN2 at different times. Proportional relationship among larval mortality, time with (**a**). Spore (**b**). ME and (**c**). MF8 doses.

Therefore, there is a proportionate relationship among spore concentration, larval mortality and treatment time (Fig. 6a).

### Larvicidal activity of the methanolic extracts (ME) of *T. asperellum* (TaspSKGN2)

Fungal extracts of *T. asperellum* (TaspSKGN2) were extracted in methanol from fully grown fungi on the most effective natural medium i.e. wheat seed medium, for treatment. Five doses (0.02, 0.04, 0.06, 0.08, 0.10 mg/mL) were prepared from lyophilized methanolic powder. No larval death was observed at a dose of 0.02 mg/mL within 1 h. Larval mortality (%) was noted after 5 h of treatment i.e., 3.33 ± 2.88 and mortality was increased with increasing time i.e., 8.33 ± 2.88 and 11.66 ± 2.88% larval mortality was found at 10 and 15 h, respectively. At a dose of 0.04 mg/mL, larval mortality was noted at 5 h i.e. 11.66 ± 2.88% and mortality increased following same trend observed before i.e., 21.66 ± 2.88 and 33.33 ± 2.88% larval mortality was observed at 10 and 15 h, respectively. Then 6.66 ± 2.88% larval mortality was noted at 1 h at the dose of 0.06 mg/mL. With this dose, 23.33 ± 2.88, 28.33 ± 2.88 and 41.66 ± 7.63% mortalities were found at 5, 10, and 15 h, respectively. At a dose of 0.08 mg/mL, 16.66 ± 5.00% and 43.33 ± 7.63% larval mortalities were observed at 1 and 5 h respectively. More than 50% of the larval mortality was found with this dose after 10 h of treatment i.e. 53.33 ± 7.63% and more than 60% of the larval mortality occurred at 15 h i.e., 61.66 ± 10.40%. At a dose of 0.10 mg/mL approximately 30% of the larval mortality was found at 1 h i.e., 28.33 ± 5.77% and more than 60 (66.66 ± 5.77) and 80% (81.66 ± 5.77) of the larval deaths were observed at 5 and 10 h, respectively. At this dose, 100% larval death was found after 15 h of treatment.

The LD_50_ value was calculated using regression analysis with MS EXCEL. The LD_50_ value of ME of TaspSKGN2 was 0.073 mg/mL (Table 4).

**Table 4:** LD_50_ calculation using regression analysis with MS EXCEL.

Isolate	Regression equation	R^2^ value	LD_50_ value (mg/mL)
TaspSKGN2	Y = 891.65X + (−14.837)	0.940	**0.073**

The LD_50_ dose of ME of TaspSKGN2 was applied to the anopheline larvae to determine the lethal time. Every set was run in triplicate. Then, 13.33 ± 5.77% larval deaths were noted after 1 h of treatment. Mortality increased with increasing time. More than 30% of the mortality was observed at 5 h i.e., 31.66 ± 7.63 and approximately 50% of the larval mortality was observed after 10 h of treatment i.e., 48.33 ± 2.88. A total of 58.33 ± 7.63% larval mortality was observed at 15 h.

The ME of TaspSKGN2 exhibited an LT_50_ value of 11:53 h. The value was calculated using regression analysis with MS EXCEL (Table 5).

**Table 5:** LT_50_ calculation of the ME of TaspSKGN2 with MS EXCEL.

Isolate	Regression equation	R^2^ value	LT_50_ value (h)
TaspSKGN2	Y = 3.20X + 13.099	0.971	**11.53**

From our results, we noted that there was a proportional relationship among larval death, ME dose concentration and time (Fig. 6b).

### Column chromatographic separation of crude ME and *in vitro* trial against anopheline larvae with fractions

Column chromatography was performed to fractionate the methanolic extract of TaspSKGN2. Among the twelve fractions, MF8 exhibited the most lethal effect on anopheline larvae.

### Larvicidal activity of MF8 of *T. asperellum* (TaspSKGN2)

After column chromatographic separation of the crude ME of *T. asperellum* (TaspSKGN2), twelve methanolic fractions (MF1-MF12) were examined for activity against anopheline larvae to detect the effective fractions, (data not given) after lyophilization. The MF8 showed the best larvicidal efficacy while MF1-MF4 exhibited no efficacy. The MF8 data are presented here. Five doses were prepared from the lyophilized methanolic powder of MF8. No larval death was observed at a dose of 0.02 mg/mL within 1 h. Larval mortality was noted after 5 h of treatment i.e., 5% and at 10 and 15 h, the mortality increased with increasing time i.e., 11.66 ± 2.88 and 18.33 ± 2.88%, respectively. At a dose of 0.04 mg/mL larval mortality started at 1 h i.e., 3.33 ± 2.88% and mortality increased following the same trend at 5, 10 and 15 h i.e. 18.33 ± 2.88, 28.33 ± 2.88 and 41.66 ± 5.77%, respectively. A mortality of 13.33 ± 2.88% was observed at 1 h at a dose of 0.06 mg/mL. At 5, 10 and 15 h, the mortality was 30 ± 5, 48.33 ± 2.88 and 61.66 ± 7.63%, respectively. At a dose of 0.08 mg/mL, 23.33 ± 2.88 and 46.66 ± 7.63% larval mortality were observed at 1 and 5 h, respectively. More than 60% of the larval mortality for this dose was found after 10 h of treatment i.e., 68.33 ± 7.63% and more than 80% of the larval lethality occurred at 15 h i.e. 85 ± 5%. At a dose of 0.10 mg/mL more than 40% of the larval death was found at 1 h i.e., 45 ± 13.22% and more than 70% (75 ± 5.00) and 90% (96.66 ± 5.77) of larval death were observed at 5 and 10 h, respectively. At this dose, 100% larval mortality was found after 15 h of treatment. The LD_50_ value was calculated using regression analysis with MS EXCEL. The LD_50_ value of MF8 of TaspSKGN2 was 0.059 mg/mL (Table 6).

**Table 6:** LD_50_ calculation using regression analysis with MS EXCEL.

Isolate	Regression equation	R^2^ value	LD_50_ value (mg/mL)
TaspSKGN2	Y = 1050X + (−12.338)	0.990	**0.059**

An LD_50_ dose of MF8 (0.059 mg/mL) of TaspSKGN2 was applied on anopheline larvae to determine lethal time. Every set was run in triplicates. Then, 16.66 ± 2.88% of larval deaths was observed after 1 h of treatment. Mortality increased with increasing time. More than 35% of the mortality was observed at 5 h i.e., 38.33 ± 5.77 and more than 50% of the larval mortality was observed after 10 h of treatment i.e. 56.66.33 ± 5.77. A total of 75% (75 ± 5) of the larval mortality was observed at 15 h. MF8 of TaspSKGN2 exhibited an LT_50_ value of 8.57 h. The value was calculated using regression analysis with MS EXCEL (Table 7).

**Table 7:** LT_50_ calculation of MF8 of TaspSKGN2 with MS EXCEL.

Isolate	Regression equation	R^2^ value	LT_50_ value (h)
TaspSKGN2	Y = 4.09X + 14.93	0.991	**8.57**

From our experiment we noted that there was a proportional relationship among larval death, MF8 dose concentrations and time (Fig. 6c).

### Thin Layer Chromatographic study of MF8

**Figure 7:**
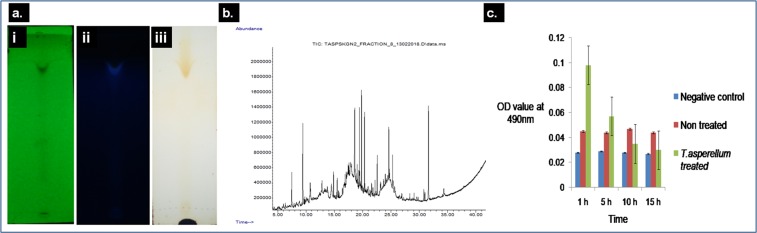
Thin layer chromatography and GC-MS study of fungal ME, and its effect in reduction of the phenoloxidase inside the anopheline larvae. (**a**) Spots of the MF8 of *T. asperellum* (TaspSKGN2) in TLC plates under UV (254 nm & 366 nm) and within an iodine vapor chamber. (*Rf* value: 0.83) [i. Spot under UV at 254 nm ii. Spot under UV at 366 nm iii. Spot within Iodine vapour chamber]. (**b**) GC-MS chromatogram of the MF8 of *T. asperellum* ME extracts exhibiting peaks of 49 compounds. (**c**) Graphical representation of the reduction in phenoloxidase activity after the initial increase inside the anopheline larvae (N = 10) treated with the LD_50_ dose of ME of *T. asperellum* in different interaction times.

The MF8 of TaspSKGN2 exhibited single spot on the TLC plates under UV (250 nm & 366 nm) and in iodine vapor (Fig. 7a). The calculated *R*_*f*_ value (Retardation factor) of MF8 was 0.83. The *R*_*f*_ value is a physical constant that can be used to identify compounds. Initially we tried to detect the compounds present in MF8 and we obtained a single spot, where the mobile phase was methanol:hexane (7:3). The spot may represent a mixture of different compounds that have same affinity to our used solvents.

### GC MS analysis of fraction 8 (MF8) of TaspSKGN2

GC MS analysis of fraction 8 exhibited peaks of 49 compounds (Table 8) (Fig. 7b). These compounds were identified with the NBS library database. (National Burea of Standard, USA)^30^. Among the 49 compounds, 11 compounds showed peak area ≥1% and ≥50% matching quality in library search (Table 9). These compounds were as follows: Benzeneacetaldehyde, 2,3-dihydro-3,5-dihydroxy-6-methyl-4H-Pyran-4-one-(7.23%), 2,3-dihydro-thiophene, (1.82%), 4,4-Dimethyl-2-cyclopenten-1-one (1.69%), p-cymene (7.19%), alpha-pinene (5.32%), hexadecanoic acid (1.12%, 1.43%), (Z,Z)-9,12-Octadecadienoic acid, methyl ester (4.62%, 2.35%) and 1,2,3,4,4a,9,10,10a-octahydro-7-methoxy-1,1,4a-trimethyl-8-(1-methylethyl)-,(4aS-trans) phenanthrene (10.22%). Seventeen compounds exhibited ≥1% peak area but scored below 50% matching quality in the library search (Suppl. Table 12). These compounds were as follows: 2-methyl piperidine (1.63%), 5-methyl-3-heptene (1.80%), 2-methylpropyl ester hexanoic acid (4.55%), decahydro-2,6-dimethyl- naphthalene (2.54%), 2-methyl-5-(1-methylethyl)-Bicyclo[3.1.0]hex-2-ene, (1.04%), hexahydro-2-methyl-7-p-tolyl-1,2-Oxazepine (1.72%), (dichloro methylene) bis[trimethyl-silane, (1.11%), (2-methylbutyl)-benzene (4.92%), 8-methyl-quinoline (4.38%), [1R-(1.alpha.,4a.beta.,7.beta.,8a.alpha.)]-decahydro-1,8a-dimethyl-7-(1-methylethyl)-naphthalene, (1.47%), 5H-Indeno[1,2-b]pyridine (1.84%), didehydro 4-methyl-1-(1-methylethyl)-. Bicyclo[3.1.0]hexane, derive.b (5.65%), 1,2-dioctylcyclopropene (3.76%), methyl 1-(propylthio)ethyl disulfide (1.12%), 2,5-dibromo-3,4-Hexanedione (1.20%), silver(1+)salt dodecanoic acid, (1.02%), 1,2-dichloro-3-nitro-benzene (1.43%). Each compound was subjected to a bio activity search. Seven compounds with high abundance were found to have potent insecticidal activity, as reported by earlier studies. These compounds were 2,3dihydro-thiophene (1.82%), 8-methyl quinoline (4.38%), p-cymene (7.19%), alpha-pinene (5.32%), hexadecanoic acid (1.12%, 1.43%), (Z,Z)-9,12-Octadecadienoic acid, methyl ester (4.62%,2.35%), 2,3-dihydro-3,5-dihydroxy-6-methyl- 4H-Pyran-4-one, (7.23%) (Table 10).

**Table 8:** Compounds identified in the MF8 of TaspSKGN2 by library search.

Sr. No.	Retention Time	Area %	Compound Name	Ref.	CAS NO.	Quality/Probability (%)
1	7.470	3.08	Benzeneacetaldehyde	64545	000122-78-1	91
2	9.426	7.23	2,3-dihydro-3,5-dihydroxy-6-methyl-4H-Pyran-4-one	66272	028564-83-2	72
3	9.763	0.37	3,4-dihydro-1-methyl-4-thioxo-2(1H)-Pyrimidinone	7787	035455-86-8	27
4	10.650	0.83	2-(diethylamino)-Ethanol	64392	000100-37-8	47
5	10.728	1.82	2,3-dihydro-thiophene	673	001120-59-8	65
6	10.785	1.63	2-Methylpiperidine	63286	000109-05-7	30
7	12.804	1.80	5-Methyl-3-heptene	2641	000000-00-0	47
8	14.485	4.55	Hexanoic acid, 2-methylpropyl ester	68344	000105-79-3	27
9	14.858	1.69	4,4-Dimethyl-2-cyclopenten-1-one	2291	022748-16-9	50
10	15.502	2.54	Naphthalene,decahydro-2,6-dimethyl-	14151	001618-22-0	42
11	15.720	0.58	Azetidine, 1-chloro-2-phenyl-	14257	030839-64-6	35
12	16.420	0.63	Benzenebutanoic acid, 2,5-dimethyl	20735	001453-06-1	46
13	16.602	0.39	2-(diethylamino)-Ethanol	64392	000100-37-8	25
14	16.664	0.46	2,4-Docosanediol, 3,5-dimethyl-	51410	056324-81-3	35
15	16.706	0.13	1-Deoxy-d-altritol	13842	000000-00-0	42
16	16.903	0.80	Benzene, 1-ethyl-3,5-dimethyl-	65553	000934-74-7	25
17	17.100	1.04	Bicyclo[3.1.0]hex-2-ene, 2-methyl- 5-(1-methylethyl)-	65773	002867-05-2	16
18	17.141	0.67	Nonanoic acid	67433	000112-05-0	17
19	17.219	0.90	Mono-ethylmalonate monoamide	5596	000000-00-0	16
20	17.281	0.67	D-Ribo-Hexose, 2,6-dideoxy-3-O-methyl-	12821	013089-76-4	43
21	17.442	1.71	1,2-Oxazepine, hexahydro-2-methyl- 7-p-tolyl-	24101	003358-89-2	25
22	17.577	1.11	Silane,(dichloromethylene)bis[trimethyl-	29429	015951-41-4	17
23	17.800	0.37	6-Heptenoic acid, methyl ester	7885	001745-17-1	18
24	17.873	0.63	1,2,3,5-Cyclohexanetetrol, (1.alph a., 2.beta., 3.alpha., 5.beta.)-	9187	053585-08-3	47
25	17.899	0.10	Oxirane, decyl-	68991	002855-19-8	42
26	18.563	4.92	Benzene, (2-methylbutyl)-	9377	003968-85-2	47
27	18.605	4.38	Quinoline, 8-methyl-	66268	000611-32-5	22
28	19.014	1.47	Naphthalene, decahydro-1,8a-dimethyl-7-(1-methylethyl)-, [1R-(1.alpha.,4a.beta., 7.beta.,8a.alpha.)]-	24999	015404-63-4	38
29	19.336	1.84	5H-Indeno[1,2-b]pyridine	14322	000244-99-5	22
30	19.435	7.19	Benzene, 1-methyl-4-(1-methylethyl)/p-Cymene	65539	000099-87-6	65
31	19.798	5.65	Bicyclo[3.1.0]hexane, 4-methyl-1-(1-methylethyl)-, didehydro deriv.	6650	058037-87-9	35
32	20.327	5.32	alpha.-Pinene	65808	000080-56-8	67
33	20.384	0.56	3-Penten-2-ol	62850	001569-50-2	27
34	21.578	1.12	Hexadecanoic acid	72011	000112-39-0	93
35	21.816	0.96	4-Decene, 8-methyl-, (E)-	11078	062338-50-5	37
36	22.190	1.43	Hexadecanoic acid	71609	000057-10-3	64
37	22.569	3.76	1,2-Dioctylcyclopropene	36682	001089-40-3	38
38	23.119	0.76	Disulfide, methyl 1-(propylthio)ethyl	18177	069078-87-1	25
39	23.186	0.81	Thiophene, 2-butyl-5-ethyl-	14618	054411-06-2	14
40	23.700	0.62	Oxirane, 2-(chloromethyl)-2-phenyl	14442	001005-91-0	22
41	23.996	1.12	Disulfide, methyl 1-(propylthio)ethyl	18177	069078-87-1	37
42	24.457	1.20	3,4-Hexanedione, 2,5-dibromo-	37620	039081-91-9	16
43	24.618	4.62	9,12-Octadecadienoic acid (Z,Z)-, methyl ester	72617	000112-63-0	99
44	24.727	1.02	Dodecanoic acid, silver(1+) salt	43821	018268-45-6	47
45	25.288	2.35	9,12-Octadecadienoic acid (Z,Z)-	72248	000060-33-3	91
46	30.819	1.43	Benzene, 1,2-dichloro-3-nitro-	20404	003209-22-1	35
47	31.005	0.80	5.alpha.-Estran-2-one	35950	004967-97-9	30
48	31.623	10.22	1,2,3,4,4a,9,10,10a- octahydro-7-methoxy-1,1,4a-trimethyl-8-(1-methylethyl)-, (4aS-trans) phenanthrene	72786	015340-83-7	74
49	34.337	0.70	Cyclotrisiloxane, hexamethyl-	27918	000541-05-9	32

**Table 9:** Eleven compounds in the MF8 of the ME of TaspSKGN2, each having a > 1% peak area and a ≥ 50% match quality in NBS library search.

Sr. No.	Retention Time	Area %	Compound Name	Ref.	CAS NO.	Quality/Probability (%)
1	7.470	3.08	Benzeneacetaldehyde	64545	000122-78-1	91
2	9.426	7.23	2,3-dihydro-3,5-dihydroxy-6-methyl-4H-Pyran-4-one	66272	028564-83-2	72
3	10.728	1.82	2,3-dihydro-thiophene	673	001120-59-8	65
4	14.858	1.69	4,4-Dimethyl-2-cyclopenten-1-one	2291	022748-16-9	50
5	19.435	7.19	Benzene, 1-methyl-4-(1-methylethyl)/p-cymene	65539	000099-87-6	65
6	20.327	5.32	Alpha pinene	65808	000080-56-8	67
7	21.578	1.12	Hexadecanoic acid	72011	000112-39-0	93
8	22.190	1.43	Hexadecanoic acid	71609	000057-10-3	64
9	24.618	4.62	(Z,Z)-9,12-Octadecadienoic acid, methyl ester	72617	000112-63-0	99
10	25.288	2.35	(Z,Z)- 9,12-Octadecadienoic acid	72248	000060-33-3	91
11	31.623	10.22	1,2,3,4,4a,9,10,10a- octahydro-7-methoxy-1,1,4a-trimethyl-8-(1-methylethyl)-, (4aS-trans) phenanthrene	72786	015340-83-7	74

**Table 10:** Reported insecticidal activity of seven compounds having high abundance in the MF8 of the ME of TaspSKGN2.

Sr no.	Peak no.	Retention time	Percentage of peak area (%)	Compounds name	Molecular Formula	Molecular Weight	Bio Activity
1	2	9.426	7.23	2,3-dihydro-3,5-dihydroxy-6-methyl-4H-Pyran-4-one	C_6_H_8_O_4_	144.1253 g/mol	Insecticidal^45^
2	30	19.435	7.19	p-cymene	C_10_H_14_	134.2182 g/mol	Mosquito Larvicidal^46^
3	32	20.327	5.32	Alpha pinene	C_10_H_16_	136.238 g/mol	Insecticidal^47^
4	27	18.605	4.38	8-methyl quinoline	C_10_H_9_N	143.19 g/mol	Mosquito Larvicidal^48^
5	43	24.618	4.62	(Z,Z)-9,12-Octa decadienoic acid,methyl ester	C_18_H_32_O_2_	280.445 g/mol	Mosquitocidal^49^
6	5	10.728	1.82	2,3-dihydro- thiophene	C_4_H_6_S	86.152 g/mol	Herbicidal, Pesticidal, Insecticidal^50^
7	36	22.190	1.43	Hexadecanoic acid	C_16_H_32_O_2_	256.43 g/mol	Nematicidal, Pesticidal, Potent mosquito larvicidal^51^

### Detection of phenoloxidase activity

The LD_50_ dose of the ME (0.073 mg/mL) was applied to anopheline larvae (n = 10); in the 1 h treatment set, the larval homogenate exhibited a higher OD value (0.098) than the non treated larval homogenate (0.045). Therefore, an initial increase in the phenoloxidase content was observed. From the 5 h treatment set, the larval homogenate showed a slightly higher OD value (0.057) than the OD value of non treated (0.044) larval homogenate but lower than the OD value of the larval homogenate from the 1 h treatment set (0.098). However, after 10 h of treatment, the larval homogenate exhibited a lower OD value (0.035) than the OD value of the non treated larval homogenate (0.047) and after 15 h of treatment, the larval homogenate exhibited the lowest OD value (0.030) i.e., near about the OD value of negative control (blank) (0.027).Our results clearly indicated that phenoloxidase content in the larval haemolymph and cuticle sharply decreased after the initial increase and treatment with the fungal ME interacted with time. (Fig. 7c).

### Microscopic study of host-pathogen interaction

**Figure 8:**
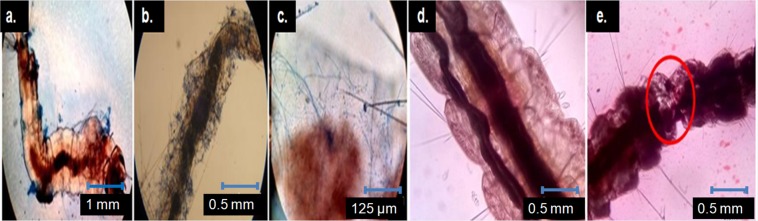
*T. asperellum* (TaspSKGN2) spores and ME treated dead *Anopheles* 3^rd^ instar larvae (microscopic view). (**a**) TaspSKGN2 spores (lactophenol cotton blue stained) attached on the outer body surface and blocking spiracles of the treated larvae (4x). (**b**,**c**) Hyphal outgrowth from the inner surface of the infected larvae (10x and 40x, respectively]. (**d**) Non treated larvae stained with alizarine (10x) e. Tissue degeneration of the ME treated larvae (red marked area) stained with alizarine (10x).

A microscopic study of the fungal spore treated dead larvae revealed that the fungal spores of TaspSKGN2 mainly blocked the spiracles of the anopheline larvae (Fig. 8a) during their entry through these openings. Fungal spores were found to be attached on outer body surfaces of the larvae at different places (Fig. 8a). The growth of the fungal hyphae from the inner side of the larval body was clearly visible.(Fig. 8b,c). A microscopic study of the ME (TaspSKGN2) treated dead larvae showed internal tissue degeneration of the larvae (Fig. 8e).

## Discussion

With the help of morphological characteristics and molecular data (ITS 1-5.8S-ITS2), we identified the fungal isolate as *Trichoderma asperellum* TaspSKGN2. Furthermore, based on the molecular data, we constructed a phylogenetic tree as taxonomy & phylogeny-based systematics are now more reliable^31–33^.

In our experimental study *T. asperellum* showed promising potential in the control of the larvae of *Anopheles spp*. alone as per our experimental results. Therefore, in the context of increasing resistance in mosquitoes to synthetic insecticides and increasing environmental pollution, our study may provide an eco friendly, green approach that may eventually play an essential role in successful mosquito control programs. Furthermore, this is the first report on the entomopathogenicity of *T. asperellum* and its use as mosquito larvicides. Our novel work may influence the research direction of other *Trichoderma* i.e., using *Trichoderma* as an entomopathogen. In our experiments, spores of TaspSKGN2 exhibited LD_50_ value of 2.68 × 10^7^ conidia/mL and LT_50_ value of 12.33 h against anopheline larvae. In this context, study of earlier workers have presented in tabular form (Table 11).

**Table 11:** A comparative table of the LD_50_ and LT_50_ values of other entomopathogens against anopheline larvae as found in literature.

Entomopathogenic fungus	Target organism	LD_50_ value (Conidia/ml)	LT_50_ value (d)	Ref no.
*Beauveria* sp.	*Anopheles* sp. larvae		3.5	^68^
*Metarhizium* sp.	*Anopheles* sp. larvae		3.39	^68^
*Beauveria* sp.	*Anopheles* sp. larvae	8 × 10^10^		^69^
*Metarhizium* sp.	*Anopheles* sp. Larvae	4 × 10^10^		^69^
*Beaveria bassiana*	*Leucinodes orbonalis*	4 × 10^7^	9.77	^70^

Compared with earlier studies (Table 11), in our experiments, the LD_50_ (2.68 × 10^7^ conidia/mL) and LT_50_ (12.33 h/0.51 d) values were lower, when the larvae were treated with isolates of *Trichoderma asperellum* (TaspSKGN2).

When larvae were treated with the fungal ME of TaspSKGN2 and viewed under the microscope, it was observed that the internal tissues of the larvae had degenerated at different sites after a few seconds following larval death. This degeneration may be caused by structural integrity loss of the larval membrane and cell dehydration due to the loss of body fluid. Damaged tissues in larvae after fungal infection have been previously observed and it has been confirmed that some toxins from the fungus kill the host through the degeneration and dehydration of the host tissue^34^ which are similar findings to our observations.

To determine the possible mechanisms of action of entomopathogens to kill their host *Anopheles* larvae, some studies have performed experiments. In an earlier study, researchers reported that the floating hydrophobic conidia of entomopathogens came in contact randomly with larvae when the larvae rose from lower depths to the upper surface of the water for feeding^35^. As entomopathogenic fungi possess defined rodlet layers on the outer surface of their conidia, we can assume that the rodlet layers play an important role in spore adhesion to the larval body surface. It was also noted by some studies^36–38^, that the rodlet layers of *Aspergillus* conidia contribute to their hydrophobicity and adhesion during attachment to the host body, findings which support our statement.

In our study, when treated larvae were observed under a microscope, we observed conidial blockage of the larval spiracles and mouth due to the entry of the fungal spore into the body of the larvae through these openings, this entry constitutes a probable cause of larval death by mechanical blockage. Similarly, earlier studies have reported that the spiracles of anopheline larvae are plugged due to the entry of fungal spores, causing larval death^39,40^. The head of the mosquito larvae is an equally important fungal infection site that supports our observation^41^. Further, we also observed that a few spores were attached to the body surface of the larvae and these spores germinated and penetrated through the larval cuticle, followed by vegetative fungal growth.

Fungal hyphae may penetrate through the larval cuticle by releasing endotoxins. Toxins may lead to larval tissue damage and toxicity inside the hemocoel and guts of mosquito larvae. It has been mentioned that dry conidia of entomopathogens when ingested by mosquito larvae, may release lethal substances without germination into the gut of the larvae, causing toxicity^39,40^.

We performed fungal extraction experiments and used the extracts (in the form of methanolic extracts from grain media) to assess mortality in *Anopheles* mosquitoes. Methanolic extract exhibited larvicidal activity when applied to anopheline larvae. Since many of the compounds have been shown to act synergistically^41^, it is worth testing the cytotoxic and cell growth inhibitory effects of the whole fungal extract rather than its individual components. This principle (synergy) allows for stronger effects when used as a whole products, while quenching or nullifying any unwanted side-effects contributed to the presence of the individual components^42^. Earlier studies have observed the larvicidal activity of a dichloromethane extract of *Beauveria* mycelium^43^. The methanolic extracts were further fractionized using column chromatography and some of the fractions showed efficacy against the larvae of *Anopheles* spp. after application with water confirming the presence of the active compounds responsible for the death of mosquito larvae. Two separate studies reported the larvicidal activity of Beauvericin from *Beauveria*^44^, and the larvicidal activity of destruxins from *Metarhizium* has been mentioned^39^. We fractionized the crude ME, and each of the fractions was tested against anopheline larvae. Of the 12 fractions, MF8 was found to be most effective against anopheline larvae. Study of GC-MS of MF8 revealed the presence of 49 compounds through a library search (NBS75K.L) (Table 8). Among these, seven compounds were reported as insecticidal or mosquitocidal in earlier studies (Table 10)^45–51^. As these compounds were found in high abundance in MF8, we can conclude that the toxicity of our methanolic extracts against the anopheline larvae was caused by the synergistic effects of these insecticidal compounds.

It is an established fact that phenoloxidase (PO) is an innate immune defense of insects against microbes, but the mechanism of the interaction between fungi and mosquitoes with respect to PO activity has remained neglected thus far. The Efficacy of *B. bassiana* secondary metabolites in subverting the cellular immune response with respect to the PO activity inside *Eurygaster integriceps* has been previously described^52^, where it was observed that the fungal secondary metabolites exhibit a dose-dependent effect, because the PO activity decreased along with the increased concentrations of the fungal metabolites. PO activates hemocyte production for self defense in insects, decreased PO activity reduces insect immunity by decreasing hemocyte production and influences fungal growth inside the insect body when fungal conidia injected. Our study determining the phenoloxidase content during fungal infection reveals that the PO activity increases inside mosquito larvae initially which indicates a high immune response of the mosquito larvae against the fungal methanolic extract at the initial stage of interaction. After, the phenoloxidase content reduces with time, leading to larval death. Our experimental results support the work of earlier studies^52^, which report that fungal metabolites reduce phenoloxidase content inside insects as well as reducing insect immunity.

Although a few reports are available regarding the entomopathogenecity of different fungi against anopheline larvae in different places around the world, the effective application of the spores and ME of *Trichoderma asperellum* as an anopheline larvicide is novel. Our experimental data clearly established that *Trichoderma asperellum* is an entomopathogenic fungus. Our experimental results also indicated that the isolate of *Trichoderma asperellum* used in the present experiments is more effective than study of earlier workers. The fungal strain/isolate used in the present experiments, which displayed a high efficacy against anopheline larvae, can be developed and applied on a broad scale i.e. moving from the laboratory to mosquito control programs. Future plans for the further study of this isolate include the structural determination of the active compounds in effective fractions using FTIR and NMR analysis and extensive field trials. Our work may influence other researchers to conduct research on *Trichoderma*, pursuing a new direction.

## Methods

### Isolation and purification of entomopathogenic fungi

#### Mosquito baiting technique

Soil samples were collected from cultivated lands, bare fields of Khardaha, Sodepur and Belgharia of the North 24 parganas in the state of West Bengal. In our routine laboratory work, we found these areas to be rich in micro-flora and these areas are also adjacent to our laboratory. The baiting technique outlined by Zimmerman was followed^53^. A total of 300 g of soil samples was placed in petridish upon a blotting paper. Dead mosquitoes were collected, and for surface sterilization, each mosquito was dipped in HgCl_2_ (0.02%) for one minute and subsequently washed with sterile water. The mosquitoes were placed randomly on the soil sample inside each petridish. Five milliliters of distilled water was added in the soil. Ecah petridish was sealed with parafilm and incubated inside a B.O.D incubator (Bio-Oxygen Demand) at 28 ± 2 °C temperature. The fungi that grew upon the dead mosquitoes were isolated by a plate culture technique^54^ in PDA (Potato[40%], Dextrose [2%], Agar [2%]) medium with Rose Bengal (0.005%) and antibiotic ampicillin (2 mg/mL).

#### Soil dilution technique

The Soil dilution technique was also used to isolate the entomopathogenic fungi from soils^55^. One (1) g of each soil sample was weighed. Culture tubes containing 9 mL of autoclaved distilled water were taken, Soil samples (1 g) were poured into culture tubes, marked as stock and mixed vigorously by shaking. Then the stock sample was serially diluted from 10^−1^ to 10^−6^ by micropipette into separate culture tubes. A total of 100 µL of diluted soil suspensions and stocks was placed in separate petridishes containing PDA media with Rose Bengal (0.005%) and antibiotics (2 mg/ml) by micropipette and were spread with a glass spreader. The petridishes were sealed with parafilm and incubated inside a B.O.D at 28 ± 2 °C. Fungi that grew in different dilution plates and stocks were isolated separately and purified by single hyphal tip method^55^ in PDA containing slants.

### Identification of isolated fungi

#### Phenotypical identification

Isolated fungi were identified on the basis of phenotypic characteristics (cultural characteristics i.e., colony morphology and hyphal growth pattern, and microscopic characteristics i.e. spore’s shape, size and color, structure of hyphae and conidiophore) based on literature guidelines^22–26^.

#### Molecular Identification

We have identified the potent entomopathogenic isolate of the fungus on the basis of the ITS region of the r-DNA as outlined in other studies^56–58^.

#### Extraction of genomic DNA

The genomic DNA of *Trichoderma asperellum* (TaspSKGN2) was extracted following a modified CTAB method^27^. The bands of DNA were observed by electrophoresis in a 1% agarose gel stained with ethidium bromide (EtBr) under UV light.

#### PCR of the ITS1-5.8S- ITS-2 of the r DNA

The Genomic DNA of *T. asperellum* was extracted, and the internal transcribed spacer (ITS1-5.8S-ITS2) regions were amplified by PCR (Polymerase Chain Reaction) technology using a Thermal cycler (Bio-Rad, Model no. T100). The DNA amplification reagent kit manual (GeNei, Bangalore, India) was followed. Fungus specific forward primer ITS-1 F (CTTGGTCATTTAGA GGAAGTAA)^59^ and the reverse primer ITS-4 R (TCCTCCGCTTATTGATA-TGC)^60^ were purchased from Eurofins Genomics Pvt. Ltd., Bangalore, India and used. PCR was carried out following a protocol, modified from Gardes and Bruns^59^.

#### Gene Sequencing

Sequencing of the PCR product was carried out in the Sci Genome Labs Pvt. Ltd, Kerala, India, and the sequence obtained was submitted to NCBI GenBank. The sequence was analyzed using BLAST tools^61^ provided by NCBI and an accession number was obtained.

#### Multiple sequence alignment (MSA)

MSA was performed with MEGA 7 software^62^ with the muscle alignment method, which aligned with our submitted query sequence with the query sequence of another *T. asperellum*^63^, the query sequence of the known entomopathogen, *B. bassiana*^64^ and the query sequences of a macrofungi, *Calocybe indica*^65^.

#### Phylogenetic tree construction and analysis

The MSA was obtained and subjected to phylogenetic analysis using MEGA 7 software. The neighbor-joining phylogenetic tree was constructed using the UPGMA method^29^ to infer the evolutionary position of our isolate.

#### Mass production of spore

Five types of natural media were used for mass production of spore:

Each sample (corn grain, wheat grain, paddy seeds, rice husk and sugarcane bagasse) was weighed and 50 g of each was separately mixed with 30 mL of distilled water in 250 mL conical flasks. Each medium was sterilized by autoclave. Isolates of *T. asperellum* were inoculated in these media with a 5-mm diameter fungal cutting made inside hood with a Laminar Air Flow with a cork borer from the fully grown fungi in petri dishes. Every medium with inoculum was incubated in a B.O.D incubator at 28 ± 2 °C. After 15 d of inoculation, 100 mL of sterile water was poured into each conical flasks and vortexed vigorously to dislodge the spores. Spore suspensions were collected in 250 mL reagent bottles. Spores were counted using a hemocytometer. The most effective medium, in which the fungus produced highest number of spores within 15d, was selected for further experiments.

#### Methanolic extraction from fungi grown on wheat grain

The most effective natural medium i.e., wheat grain medium, was prepared in 250 mL conical flasks with 50 g of wheat grain and 30 mL of distilled water each. *T. asperellum* (TaspSKGN2) was inoculated, and flasks were incubated at B.O.D for 15 d. Total grain with fully grown fungus was removed and kept in 1 L of conical flask. Then, 500 mL of methanol (70%) was poured at a ratio of 1:10 (grain(W):solvent(V) ratio) into the each conical flask, and the flasks were kept in a shaker for 3 d. Then the solutions were filtered and re filtered with Whatman filter paper No: 4. The filtered methanolic extract was kept in the 250 mL conical flasks and the methanol was evaporated with a vacuum evaporator (BUCHI, R 100) and lyophilized to a powder by lyophilizer (HETO F.D. 1.0). Lyophilization was performed by rapid freeze drying at −55 °C followed by sublimation (drying) under vaccum conditions.

#### Collection and identification of the larvae of *Anopheles* mosquitoes

Extensive surveys were conducted in various parts of Debagram (Nadia), Kaliachalk (Malda), Sreerampur (Hooghly), Khardaha (North 24 Parganas), South 24 Parganas and Lavpur (Birbhum) in the state of West Bengal. The majority of the mosquito larvae were found in Debagram (Latitude: 23.7333413, Longitude: 88.2333253) and Lavpur (Latitude: 23.8161° N, Longitude: 87.7985° E). Larvae were collected from different larval habitat i.e., blocked drains, stored rain water in roadside puddles, grassy ditches and water pools, with the help of small sieves and were kept in mosquito rearing tub for experimental purposes. The larvae were identified on the basis of morphological and ecological parameters with the help of a published pictorial key^66^ and identification was confirmed by a scientist from the Zoological Survey of India, Kolkata, West Bengal, through personal communication.

#### Fungal spore, ME and ME fractions treatment *in vitro* experiments

The anopheline larvae from different mosquito habitats were subjected to different treatments in this experiment. The spore suspensions were serially diluted 10 fold at different spore concentrations (10^1^–10^8^ conidia/mL) in 100 mL beakers. A total of 20 mL of spore suspension of each concentration was poured into the beaker. Five doses of lyophilized crude methanolic powder and MF8 powder of *T. asperellum* i.e., 0.02, 0.04, 0.06, 0.08 and 0.10 mg/mL were made with double distilled water and poured into different 100 mL beakers with a final total volume of 20 mL of distilled water and each dose of crude ME or MF8 solution per beaker. Twenty anopheline larvae were released into each beaker. A total of 200 mg of wheat flour was added to each beaker as larval food. In our laboratory, we have previously observed that larvae (n = 20) supplied with wheat flour (200 mg) can survive within beakers in water, and their life cycle was not hampered.(Unpublished). The mouths of the beakers were sealed with perforated polyethylene sheets, and the beakers were kept at room temperature (30 °C) inside the laboratory. The results of the interaction between the anopheline larvae and the spore suspensions of *T. asperellum* (TaspSKGN2) were taken at 24 h intervals, and the interactions between the anopheline larvae and the crude ME (TaspSKGN2) as well as the anopheline larvae and MF8 were monitored at one hour intervals.

#### LD_50_ and LT_50_ determination using statistical analysis

Each experiment was performed in triplicate with controls, and the mean value of the mortality rate was taken for analysis. The LD_50_ (lethal dose for 50% mortality) and LT_50_ (lethal time for 50% mortality) values of each isolate were calculated by regression analysis with MS EXCEL; the mortality was determined based on the straight line equation [Y = aX + b]. [where X = independent variables (Spore/metabolites concentrations/time intervals of observations); Y = dependent variables (larval death percentages), n = number of frequencies; b = regression co-efficient].

#### Fractionization of the methanolic extract by Column Chromatography and the effect of fractions on larvae

A silica gel column (100–200 mesh) (5 ft.) was packed with methanol and hexane at a ratio of 7:3. Lyophilized powder of TaspSKGN2 was dissolved in methanol solvent and charged in silica gel column. The fractions were collected in microcentrifuge tube (2 mL). Twelve fractions (MF1-MF12) were collected. Each fraction (2 mL) was evaporated in vacuum evaporator and the residue powder was re dissolved in 20 mL of double distilled water in a 100 mL beaker. The effect of the each fractionon the anopheline larvae was recorded as described previously.

#### Thin Layer Chromatographic study of MF8

The most effective fraction (MF8) from *T. asperellum* was dissolved in methanol and run through a TLC plate (60 meshes) by applying a spot through a capillary. The mobile phase was methanol and hexane in a 7:3 ratio. Spots were visualized in UV (250 nm, 366 nm) and Iodine vapour chamber. *R*_*f*_ *value* (Retardation factor; The *R*_*f*_ value is a physical constant that can be used to identify compounds.) of each spot was calculated by the following formulae:

$${R}_{f}value=:\frac{Distance\,covered\,by\,the\,sample\,in\,the\,TLC\,plate}{Distance\,covered\,by\,the\,solvent\,in\,the\,TLC\,plate}$$

#### Gas Chromatography-Mass spectrometry (GC-MS) analysis of crude methanolic extract of *T. asperellum*

GC-MS was performed by using an Agilent Technologies, GC-6860N Network GC System with a 5973 inert Mass Selective Detector and the search library used was NBS75K.L^30^. The type of column used was HP- 5MS (19091S-602). Length and film thickness of the column were 30 m and 0.25 mm respectively. The oven temperature was set at 50 °C with an increase of 10 °C/min to 280 °C and then 300 °C for 10 min. Helium gas (99.999%) was used as the carrier gas with a constant flow rate of 1 mL/min. An aliquot of 2 μl of sample was injected into the column with an injector temperature at 280 °C. Ionizing energy (70ev) was used. The ion source temperature was 280 °C. The total GC running time was 50 min.

#### Detection of Phenoloxidase (PO) activity

To evaluate the reduction in phenoloxidase content inside the larval cuticle and hemolymph during *T. asperellum* infection, anopheline larvae (N = 10) were treated with the LD_50_ doses of the fungal methanolic extracts (0.073 mg/mL) in three different beakers with ten larvae in each beaker; larvae were removed at a given times (1, 5, 10 and 15 h) and crushed in phosphate buffer by mortar and pestle. Cell-free hemolymph plasma and homogenized cuticle fragment samples were prepared for analysis of phenoloxidase (PO) activity. A total of 10 µL of hemolymph plasma was prepared from anopheline larval tissues. This was diluted with 20 µL PBS (Phosphate buffer saline) and centrifugation was done at 1000 g for 5 min at 4 °C to remove the hemocyte pellet. Whole cleaned integument was dissected in PBS; washing was performed three times by gentle vortexing in PBS for 1 min. After washing, it was homogenized in 200 µL PBS for 2 min with homogenizer. After that the homogenate was centrifuged for 15 min at 12000 g at 4 °C. The supernatant of both the hemolymph plasma and the cuticle was used for spectrophotometric analysis of PO enzymatic activity and protein concentration in a modification of the Lowry’s method. Concentration of PO was estimated using L-DOPA (L-3,4-dihydroxyphenylalanine) as substrate which reacts with insect PO and produces quinone as an end product which colour is brown. More intensity of the brown colour indicated the increased concentration of PO and vice-versa. 10 µL aliquots of plasma, 50 µL of the cuticle homogenate were added to the wells of a flat-bottomed 96-well microtitre plate containing 200 µL of 10 mM L-DOPA dissolved in sterile pyrogen-free water. After 30 min incubation at 28 °C, the absorbance was quantified at 490 nm using a plate reader (BIO-RAD, i mark). Fresh healthy larval homogenates were used as control. A blank was set up with distilled water replacing anopheline larval homogenates (negative control). PO experiments were conducted in triplicates The PO activity was expressed as the change in absorbance at 490 nm.

#### Microscopic determination of mortality

Dead larvae treated with fungal spores were stained with lactophenol cotton blue (Sigma-Aldrich, Density: 1.16 g/mL at 20 °C) on grease free slides to detect the fungal spore attachment and the presence of germinating hyphae. Fungal ME treated dead larvae were stained with alizarin (0.02%) (Sigma-Aldrich, 97%), to stain the alimentary system of the dead anopheline larvae for the detection of the effect of fungal ME on the larval internal tissue system; cover slips were added to the grease free slides over the sample and gently pressed flat with fingertip and the slides were observed under a compound microscope (Olympus CX31)^67^ at different magnifications (4x, 10x and 40x).

## Supplementary information

A new application of Trichoderma asperellum as an anopheline larvicide for eco friendly management in medical science.

## Data Availability

Data are available from www.nature.com.
