# Supplementary material for: A new application of Trichoderma asperellum as an anopheline larvicide for eco friendly management in medical science
Source: Sci Rep. 2019 Feb 1;9:1108. doi: 10.1038/s41598-018-37108-2 (PMC6358612; doi:10.1038/s41598-018-37108-2)
Supplement: Supplementary file 1 — A new application of Trichoderma asperellum as an anopheline larvicide for eco friendly management in medical science. [file 41598_2018_37108_MOESM1_ESM.docx]

**A new application of *Trichoderma asperellum* as an anopheline larvicide for eco friendly management in medical science.**

**Dipanjan Podder & Swapan Kr. Ghosh***

**Molecular Mycopathology Lab., Biological Control and Cancer Research Unit, PG Department of Botany, Ramakrishna Mission Vivekananda College (Autonomous), Rahara, Kolkata-700118, W.B., India.**

***Corresponding author: gswapan582@gmail.com**

**Suppl. Table 12.** Seventeen compounds in F8 of ME of TaspSKGN2 having >1% peak area

and < 50% matching quality in library search.

| Sr.  No. | Retention Time | Area (%) | Compound Name | | Ref. | | CAS NO. | | Quality | |
| --- | --- | --- | --- | --- | --- | --- | --- | --- | --- | --- |
| 1 | 10.785 | 1.63 | | 2-Methyl  piperidine | | 63286 | | 000109-05-7 | | 30 |
| 2 | 12.804 | 1.80 | | 5-Methyl-3-heptene | | 2641 | | 000000-00-0 | | 47 |
| 3 | 14.485 | 4.55 | | Hexanoic acid, 2-methylpropyl ester | | 68344 | | 000105-79-3 | | 27 |
| 4 | 15.502 | 2.54 | | Naphthalene,decahydro-2,6-dimethyl- | | 14151 | | 001618-22-0 | | 42 |
| 5 | 17.100 | 1.04 | | Bicyclo[3.1.0]hex-2-ene, 2-methyl- 5-(1-methylethyl)- | | 65773 | | 002867-05-2 | | 16 |
| 6 | 17.442 | 1.71 | | 1,2-Oxazepine, hexahydro-2-methyl- 7-p-tolyl- | | 24101 | | 003358-89-2 | | 25 |
| 7 | 17.577 | 1.11 | | Silane,(dichloromethylene)bis[tri  methyl- | | 29429 | | 015951-41-4 | | 17 |
| 8 | 18.563 | 4.92 | | Benzene, (2-methylbutyl)- | | 9377 | | 003968-85-2 | | 47 |
| 9 | 18.605 | 4.38 | | Quinoline, 8-methyl- | | 66268 | | 000611-32-5 | | 22 |
| 10 | 19.014 | 1.47 | | Naphthalene, decahydro-1,8a-dimethyl-7-(1-methylethyl)-, [1R-(1.alpha.,4a.beta., 7.beta.,8a.alpha.)]- | | 24999 | | 015404-63-4 | | 38 |
| 11 | 19.336 | 1.84 | | 5H-Indeno[1,2-b]pyridine | | 14322 | | 000244-99-5 | | 22 |
| 12 | 19.798 | 5.65 | | Bicyclo[3.1.0]hexane, 4-methyl-1-(1-methylethyl)-, didehydro deriv. | | 6650 | | 058037-87-9 | | 35 |
| 13 | 22.569 | 3.76 | | 1,2-Dioctylcyclopropene | | 36682 | | 001089-40-3 | | 38 |
| 14 | 23.996 | 1.12 | | Disulfide, methyl 1-(propylthio)ethyl | | 18177 | | 069078-87-1 | | 37 |
| 15 | 24.457 | 1.20 | | 3,4-Hexanedione, 2,5-dibromo- | | 37620 | | 039081-91-9 | | 16 |
| 16 | 24.727 | 1.02 | | Dodecanoic acid, silver(1+) salt | | 43821 | | 018268-45-6 | | 47 |
| 17 | 30.819 | 1.43 | | Benzene, 1,2-dichloro-3-nitro- | | 20404 | | 003209-22-1 | | 35 |

**Suppl. figure.1**


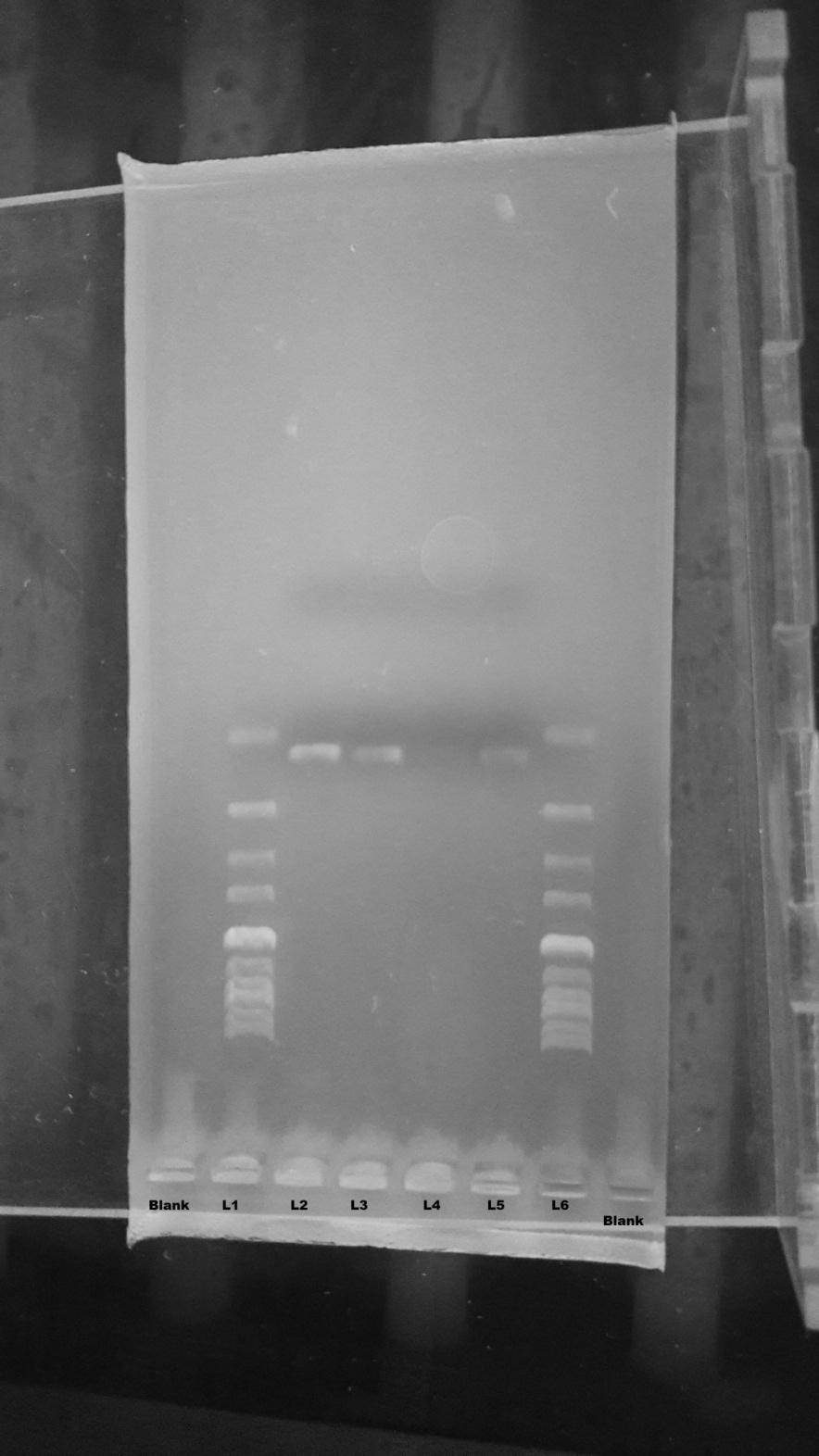


**Suppl.Figure 1. Agarose gel electrophoresis: Band pattern of PCR amplified genomic DNA under UV transilluminator. L1. DNA Ladder; L2, L3, L4 & L5. Bands of DNA amplicons .**
